# Novel interactive text-messaging curriculum for endocrinology board review

**DOI:** 10.1016/j.jcte.2023.100326

**Published:** 2023-09-29

**Authors:** Priyanka Majety, Ayodele Ajayi, Anna M. Modest, Maria Vamvini, Jason A. Freed

**Affiliations:** a Department of Endocrinology, Diabetes and Metabolism, Virginia Commonwealth University Health, Richmond, VA, United States; b Department of Obstetrics and Gynecology, Beth Israel Deaconess Medical Center, Boston, MA, United States; c Department of Obstetrics and Gynecology, Beth Israel Deaconess Medical Center, Harvard Medical School, Boston, MA, United States; d Section on Integrative Physiology and Metabolism, Joslin Diabetes Center, Harvard Medical School, Boston, MA, United States; e Division of Endocrinology, Diabetes and Metabolism, Beth Israel Deaconess Medical Center, Harvard Medical School, Boston, MA, United States; f Division of Hematology/Oncology, Beth Israel Deaconess Medical Center, Harvard Medical School, Boston, MA, United States

**Keywords:** Text-messaging, Board review, Medicine fellows, Endocrinology, ABIM exam, Exam preparation

## Abstract

**Background:**

The ABIM certification exam is one of the measures to ensure that physicians have the clinical skills for good care delivery. The 5-year average pass rate for ABIM Endocrinology exam is 82%. The pass rate significantly decreased to a nadir of 74% in 2021 and 2022, lowest of all medicine subspecialties.

**Objectives:**

To assess the feasibility of text messaging curriculum for fellows and its utility in improving their test performance.

**Methods:**

In 2021, endocrinology fellows from 51 programs across the country were invited to participate in our curriculum. They completed a pre-test, joined a texting group via Remind application and received 1 multiple choice question daily (total n = 78). After 15 weeks, they completed a post-test and survey. Paired results from pre- and post-test were compared.

**Results:**

A total of 89 fellows from 27 programs responded. Of these, 82 fellows, predominantly females (n = 60; 73 %), filled out the pre-test. On an average, 42 fellows (SD = 12) responded to the questions daily and 57 % of them answered the questions within 24 h. Thirty fellows completed the post-test. The median number of correct responses on the pre-test was 5 (IQR 3–6), compared to 8 (IQR 6–9) in the post-test. There was a significant improvement (p-value < 0.0001) in fellows’ performance in the post-test when compared with the pre-test following our intervention.

**Conclusions:**

Text-messaging based curriculum for exam preparation is feasible and can improve test performance. Fellows find receiving a daily high yield multiple choice question via text-message as a useful tool for exam preparation.

## Highlights

• A text-messaging-based curriculum for exam preparation is feasible and can improve test performance among endocrinology fellows.
• A combination of learning strategies like retrieval practices and low-stakes quizzes is an effective educational approach.
• Endocrinology fellows primarily use question banks for exam preparation but prefer mobile-based tools for more effective learning.
• They found receiving a daily multiple-choice question via text message, useful for exam preparation.

## Introduction

The American Board of Internal Medicine (ABIM) subspecialty certification exam is one of the measures to ensure that the physicians have met the expected standards and have the clinical skills for the delivery of good patient care. There is evidence that performance on these exams affects patient outcomes [1], [2], [3], [4]. The five-year average first-time taker pass rate for the ABIM Endocrinology & Metabolism subspecialty exam is 82.4 %, relatively lower when compared to other subspecialties [5]. The pass rate for first time test takers significantly decreased from a usual range of 84–91 % over 2018–2020 to a nadir of 74 % in 2021 compared to a mean of 84 % for other medicine subspecialties [6]. Similarly, in 2022 the average pass rate was 74 % compared to a mean of 91 % for other specialties. This represented the lowest pass rate in 2021 and 2022 for any internal medicine subspecialty exam.

There has been an ongoing effort to maximize educational material provided to fellows who are working in a time-constrained work environment to improve their learning during training and exam performance [6]. While question banks have been shown to improve exam performance [7], [8], wide-spread and longitudinal use of question banks during training is limited by cost and accessibility. During the COVID-19 pandemic training programs were forced to change the way education was delivered [9] and this led to educational innovations and digitalization. Mobile technology has shown promising results [10], [11], [12]. Therefore we developed a novel text-messaging curriculum for endocrinology fellows that nudges them to answer one multiple choice question daily and assessed its utility in improving their knowledge.

The primary purpose of our study was to assess the feasibility of a unique text messaging curriculum for endocrinology fellows and its utility in improving their knowledge. We also assessed their study habits and resources available to them for ABIM subspecialty exam preparation.

## Methods

We identified three topics in endocrinology (lipids/obesity, pituitary, and reproductive endocrinology) with relatively poor performance by fellows nation-wide based on the 2021 in-training exam (ITE) data [13]. With the help of experts in these fields within the Division of Endocrinology, Diabetes, and Metabolism at Beth Israel Deaconess Medical Center, we developed a pre-test questionnaire that consisted of 10 multiple choice questions. In September 2021, we emailed the program directors of 51 fellowship programs across the country (predominantly programs in the Northeast, Midwest, and Southern United States), inviting their fellows to participate in our curriculum. Written consent was obtained. Fellows were requested to complete the pre-test questionnaire and join a texting group via an application, Remind (https://www.remind.com). Fellows who signed up received one multiple choice question daily for fifteen and half weeks (five questions per week; a total of 78 questions). These questions were developed by the authors and included all the endocrinology subtopics in the ABIM blueprint [14], but with relatively higher proportion of questions in lipids/obesity, pituitary, and reproductive subtopics (Supplementary Table 1). Participants were given the option to opt out of the curriculum at any time.

**Fig. 1:**
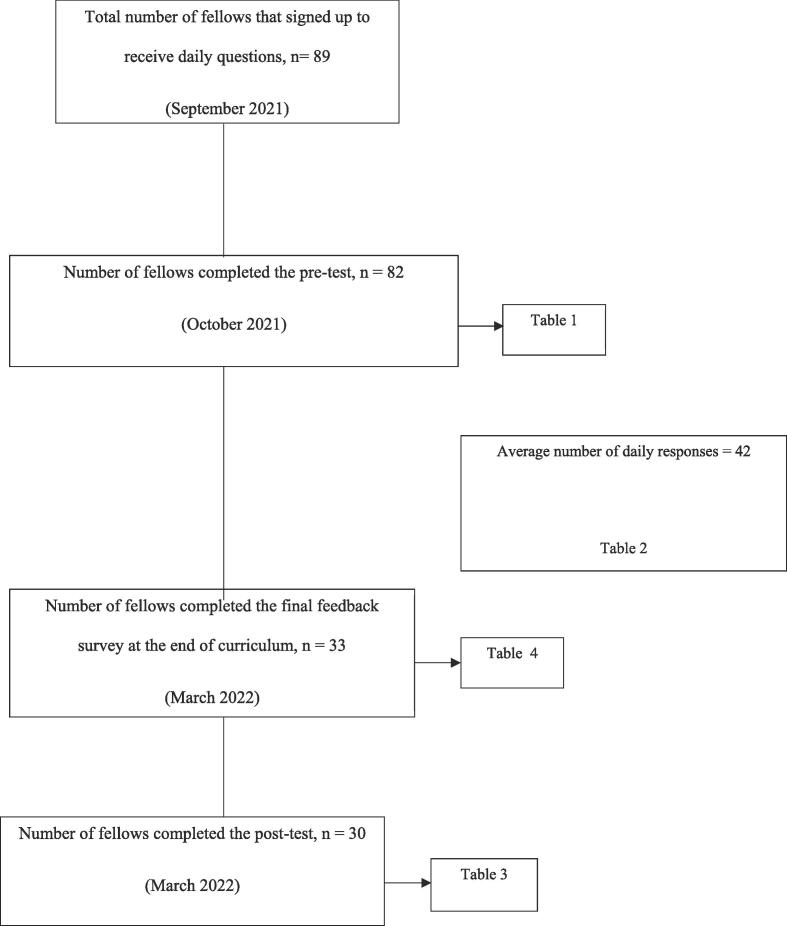
Outline and timeline of the text-messaging curriculum.

At the end of each week, a weekly summary with key learning points from the preceding week was sent to the participants. Mid-project feedback was sought from the participants after week seven to assess need for modifications of the curriculum. At the end of curriculum, fellows received a post-test questionnaire, similar to the pre-test and a final feedback survey that included questions inquiring about their study habits. Effective learning strategies such as testing with immediate feedback and retrieval practices with weekly summaries of key learning points were combined in this initiative. It did not require app downloads and there were no subscription costs, making it a flexible, cost-effective educational tool for fellows. The outline of our study is shown in Fig. 1.

All statistical tests were performed using SAS 9.4 (SAS Institute Inc., Cary, NC). All tests were two sided, and P values <0.05 were considered statistically significant. Data are presented as mean ± standard deviation (SD), median and interquartile range (IQR), or proportion. Comparisons of paired pre/post-test data were made using a Wilcoxon signed rank test. The study protocol was approved for exemption by the Beth Israel Deaconess Medical Center institutional review board.

## Results

An email was sent to program directors of 51 endocrinology programs in September 2021. A total of 89 fellows from 27 fellowship programs signed up for the curriculum. Of these, 82 fellows completed the pre-test questionnaire (Table 1). These fellows were predominantly females (n = 60; 73 %) in post-graduate year 4 (PGY-4: 46 %) or post-graduate year 5 (PGY-5: 45 %). Only 3 out of the 82 fellows (4 %) had plans to take their ABIM endocrinology board examination the same year (2021) while most of them were planning to take the exam in 1 or 2 years following our intervention (40 % in 2022 and 45 % of them in 2023). The pre-test also surveyed the participants on resources that were being used by their training programs for board exam preparation and knowledge-based, multiple-choice questions. The most common resources available to fellows beyond their fellowship didactics were board review conferences (45 %) and subscription to question banks (26 %) while only 9 % of them were provided with low-stakes quizzes by their program for board exam preparation. The post-test questionnaire was completed by 30 out of the 82 participants and these were either PGY-4 (47 %) or PGY-5 (47 %) (Table 1). There were no differences in the baseline characteristics of those who completed both the pre- and post-test questionnaire (“pre/post cohort”) compared to the overall cohort.

**Table 1:** Participant characteristics (total respondents, n = 82 and fellows who responded to both pre-and post-test, n = 30) and common resources used by fellowship programs for ABIM exam preparation.

	**Total cohort** **n = 82 (%)**	**Pre/post cohort** **n = 30 (%)**
**Gender**		
Male	22 (27)	8 (27)
Female	60 (73)	22 (73)

**Post Graduate Year (PGY)**		
PGY-4	38 (46)	14 (47)
PGY-5	37 (45)	14 (47)
PGY-6	5 (6)	1 (3)
PGY-7	2 (2)	1 (3)

**Year of boards**		
2021	3 (4)	0 (0)
2022	33 (40)	12 (40)
2023	37 (45)	13 (43)
2024	4 (5)	3 (10)
2025	2 (2)	2 (7)
Other	3 (4)	0 (0)

**Resource**		
Daily/weekly board review sessions	37 (45)	16 (53)
Low stakes quizzing (not including ESAP ITE)	7 (9)	0 (0)
Daily lectures	6 (7)	3 (10)
Weekly lectures	56 (68)	22 (73)
Subscription to Endocrine question banks	21 (26)	6 (20)
Others (please explain)*	10 (12)	2 (7)

ESAP ITE – Endocrine Self-Assessment Program In-training exam.

Data presented as n(%).

* Monthly or bi-monthly group review of Endocrine Self-Assessment Program (ESAP) questions, American Association of Clinical Endocrinology (AACE) board review course.

The participants received a total of 78 questions during the study period. On an average, 42 ± 12 participants responded to the questions daily and more than half of them answered the questions within 24 h (Table 2). The pre- and post- test included 10 knowledge-based questions. In the pre/post cohort, the median number of correct responses on the pre-test was 5 (IQR 3–6), and this significantly improved to 8 correct responses ((IQR 6–9), p < 0.01) on the post-test. Significant improvement in performance was observed in all the subtopics of the test including pituitary, reproductive endocrine, lipids and obesity (all p = <0.01) (Table 3).

**Table 2:** Patterns of responses during the curriculum.

**Topic**	**Number of questions n (%)**	**Daily responses** mean ± SD	**Correct responses** mean ± SD	**Responses within 24 h** mean ± SD
Adrenal	10 (13)	44 ± 9	27 ± 10	25 ± 6
Calcium and bone	10 (13)	43 ± 18	25 ± 14	26 ± 11
Diabetes	11 (14)	50 ± 12	33 ± 15	29 ± 9
Lipids and obesity	13 (17)	38 ± 11	20 ± 12	22 ± 8
Reproductive endocrinology	15 (19)	38 ± 11	20 ± 12	22 ± 7
Pituitary	12 (15)	40 ± 9	23 ± 11	23 ± 5
Thyroid	7 (9)	45 ± 8	29 ± 9	26 ± 7
**Total**	**78 (100)**	**42 ± 12**	**25 ± 12**	**24 ± 8**

Data presented as mean ± standard deviation.

**Table 3:** Correct answers on the pre- and post-test.

**Category**	**Pre-test** **N = 30**	**Post-test** **N = 30**	**p-value**
Pituitary (3 questions)	1 (0–2)	2 (2–3)	<0.01
Lipids and Obesity (3 questions)	2 (1–2)	3 (2–3)	<0.01
Reproductive endocrinology (4 questions)	2 (1–2)	3 (2–4)	<0.01
Total (10 questions)	5 (3–6)	8 (6–9)	<0.01

Data presented as median (interquartile range).

A mid-project feedback survey was sent to the participants after seven weeks of the intervention. Based on the positive feedback received by the majority of the participants, no changes were made to the curriculum. At the end of the curriculum, final feedback survey was distributed to the fellows which was filled out by 33 of them. The aim of this survey was to assess their study habits and board preparation preferences after having experienced our text messaging-based curriculum and subsequently evaluate the utility of this curriculum. Ninety-seven percent responded that they use question banks as a resource for exam preparation followed by PowerPoint presentations (52 %), journals (48 %), online videos (39 %), textbooks (33 %) and lecture notes (33 %). In terms of their preference regarding how they access practice questions, 23 fellows (70 %) preferred mobile friendly question bank applications, 5 fellows (15 %) preferred online question banks available through company websites only, and the remaining 5 fellows (15 %) had no preference. None of them reported preference for paper-based question banks (Table 4).

**Table 4:** Results of the final feedback survey (n = 33).

**Resources used for exam preparation**	**N = 33**
Question banks	32 (97)
Journals/articles	16 (48)
Textbooks	11 (33)
Online videos	13 (39)
Twitter	2 (6)
Lecture notes	11 (33)
PowerPoint	17 (52)
Podcasts	0
Others = UpToDate; have not started	2 (6)

**Usefulness of the curriculum**	
Not at all useful	0
Slightly useful	1 (3)
Moderately useful	3 (9)
Very useful	19 (58)
Extremely useful	10 (30)

**Preference regarding exam preparation tools**	
Paper based question banks	0 (0)
Online question banks available through company websites only	5 (15)
Mobile friendly question bank applications	23 (70)
No preference	5 (15)

**Discussion tool**	
Never	2 (6)
Rarely	3 (9)
Sometimes	17 (52)
Often	9 (27)
Always	2 (6)

**Recommend to a friend/colleague**	
Extremely unlikely	1 (3)
Unlikely	0 (0)
Neutral	0 (0)
Likely	10 (30)
Extremely likely	22 (67)

**Receive a question daily**	
Yes	32 (97)
No	1 (3)

**Barriers to participation**	
Yes	5 (28)
Hard to remember and find the time	1 (3)
Sometimes I would look and then forget to finish the question	1 (3)
Sometimes being busy on service, I would do all five questions on the weekend	1 (3)
Time	1 (3)
Busy rotations, too tired to answer one question per day. Then I forget for days	1 (3)
No	27 (82)

**Quality of the project**	
Poor	1 (3)
Fair	0 (0)
Good	2 (6)
Very good	11 (33)
Excellent	10 (58)

Data presented as n (%).

Nineteen of the 33 fellows (58 %) found the curriculum very useful, and 10 fellows (30 %) found it extremely useful. When asked if this curriculum served as a discussion tool, 17 fellows (52 %) said it served as a discussion tool sometimes, while 9 fellows (27 %) said they used it as a discussion tool often. Only two fellows (6 %) said it always served as a discussion tool and 5 fellows (15 %) said that this served as a discussion tool only rarely (n = 3; 9 %) or never (n = 2; 6 %). Thirty-two out of 33 fellows (97 %) responded that they would like to continue to receive a question daily for the rest of their fellowship. Twenty-seven fellows (82 %) had no barriers in participating in the curriculum. Six fellows (18 %) encountered some barriers in participating. Some of the common reasons being forgetting to complete the curriculum, busy schedules, and lack of time (Table 4).

## Discussion

Recently, there has been a consistent and significant decrease in the pass rate for first-time takers of the ABIM Endocrinology, Diabetes, and Metabolism certification examination. Notably, a decline in pass rates was observed across all subspecialties in 2021 during the pandemic, but this trend persisted for endocrinology in 2022. While no studies have yet delved into the reasons behind this decline, potential factors may include the COVID-19 pandemic's impact on outpatient clinics, the abrupt transition from traditional in-person didactics and conferences to digital education, and increased dependence on telehealth clinics, which resulted in reduced in-person patient care. Understanding the root causes of this sudden decline is crucial for implementing necessary curricular changes to enhance fellow education and better prepare them for the ABIM examination*.*

Mobile technology, including smartphone applications and text messaging programs, have shown promising results in education and may enhance board exam preparation [10], [12], [15], [16], [17]. This may be due to its widespread use and ease. We chose to develop a text-messaging tool instead of daily emails to avoid email fatigue in our participants [18]. Our tool distinguishes itself from other mobile question bank applications by delivering questions through text messages, actively prompting participants to respond, eliminating the need to open an application for answers. Additionally, our tool enables the convenient provision of weekly summaries with essential learning points, a feature not commonly found in other applications. It offers tailored question customization to meet the unique requirements of any fellowship program, ensuring a personalized learning experience. Importantly, our tool is cost-free, easily replicable, and does not require any subscriptions or app downloads for use. Our novel text-messaging curriculum was positively received by the fellows and led to significant improvement in test performance. We combined highly effective learning strategies, implementing retrieval practices with low stakes quizzes and weekly summary with key learning points as well as formal testing for performance assessment and feedback.

Testing is a well-studied powerful tool to help with the retention of information. While testing is most often used in the educational settings for assessment, another benefit of tests is that they improve memory of the tested information. Testing forces one to elaborate and make connections to prior knowledge; ultimately, it enhances later retention, a phenomenon known as the *testing effect* [19]*.* A major limitation of testing as a learning tool is test anxiety which can potentially undermine some of the learning benefits [20]. Retrieval practice is a learning strategy where the principle is that repeated practice in recalling information yields greater long-term memory retention gains. Examples include low stakes writing prompts, brief quizzes, flashcards. In our study we used low-stakes quizzes which can serve to decrease test anxiety and nullify the detrimental impact of stress on learning [21]. Our findings are in agreement with prior reports [22], and suggest that providing trainees with several low-stakes testing opportunities may help increase learning during training. Instant feedback during these retrieval practices can further enhance learning and retention of information [23], [24].

Currently, the only standardized testing tool available for assessing knowledge during fellowship training, is the Endocrine Self-Assessment Program ITE (ESAP-ITE) that occurs once a year. This has been shown to provide a strong predictive value for ABIM certification outcomes [25]. While the ESAP-ITE serves both as an assessment and a formative learning tool, its limitation is that the test takers perform under a stressful environment and do not receive immediate and specific feedback on the questions that were incorrectly answered. The feedback includes the fellow’s overall performance in comparison with peers nation-wide, and lists the topics that were incorrectly answered (and not specific questions) [13]. This is available to the fellows approximately, 4–8 weeks [26] after the test, when the memory of the questions has faded.

There is no published data on the resources used by training programs and endocrinology fellows for the ABIM subspecialty certification exam preparation. Our study reports that most fellows use question banks followed by PowerPoint presentations, journals, online videos, textbooks, and lecture notes. Although 97 % of respondents said they use question banks, only 26 % of have access to question banks through their programs. This discrepancy highlights that many fellows purchase question banks for exam preparation. There has been a concern about the relatively limited number of standardized learning tools that are available for endocrinology fellows-in-training and are not readily accessible due to the prohibitive costs, with very few exceptions [27], [28], [29], [30], [31]. Based on the responses we received in our study; most fellows prefer mobile based tools for exam preparation and our tool can be easily replicated and individualized to the needs of any training. It could also be used to help physicians in practice stay up to date with medical knowledge and not just for exam preparation.

Generational change and challenge in medical education is not uncommon. Changes in learner profiles, transference of learning patterns, increased mobile usage and digital transformation have enabled e-learning as a powerful platform. Younger generation of learners seek education that is efficient, personalized and technologically enhanced [32], [33]. While our educational tool is one of many resources that was made available to the fellows, we attribute its success to its ease of use, efficiency, and usage of effective learning strategies, which was created for the current generational learners. More professional organizations should consider developing mobile applications to make the question banks easily accessible to learners and until then, programs should consider text-messaging based curricula and other digital tools to enhance fellow education.

Our study was not without limitations. Firstly, our study had a limited number of participants. Only 51 out of the 160 ACGME accredited programs were invited to participate in our study. We were unable to include other fellowship training programs due to the significant differences between time zones and thereby the timing of the text-message, which would hamper fellows’ participation. Secondly, we were unable to retain all the participants until the end of the study period. About half of the participants who signed up (an average of 42 out of 82 participants who completed the pre-test) remained engaged throughout the study period. This may relate in part due to the timing of our study, which started in October 2021, while most our participants were not taking the ABIM certification exam until much later. This limits the generalizability of our study results. Finally, the questions developed by authors were not validated by other experts in the field.

Further research on text-messaging based tools for exam preparation should include a larger number of participants across various fields and longitudinal analyses to assess their effectiveness in terms of exam pass rates and learner satisfaction.

## Conclusions

Text-messaging based curriculum for exam preparation is feasible and can improve test performance. Most fellows find receiving a daily high yield multiple choice question via text-message as a useful tool for exam preparation.

## Disclosure statement

The authors have nothing to disclose.

## Declaration of Competing Interest

The authors declare that they have no known competing financial interests or personal relationships that could have appeared to influence the work reported in this paper.
