# Supplementary material for: Novel interactive text-messaging curriculum for endocrinology board review
Source: J Clin Transl Endocrinol. 2023 Sep 29;34:100326. doi: 10.1016/j.jcte.2023.100326 (PMC10570572; doi:10.1016/j.jcte.2023.100326)
Supplement: Supplementary Table 1 [file mmc1.docx]

**Supplementary table 1: Question distribution in our curriculum in comparison with ABIM Blueprint**

| **Topic** | **Question distribution in our curriculum (n)** | **ABIM blueprint distribution** |
| --- | --- | --- |
| Adrenal | 13% (10) | 10% |
| Calcium and bone | 13% (10) | 15% |
| Diabetes | 14% (11) | 24% |
| Lipids and obesity | 17% (13) | 12% |
| Reproductive endo | 19% (15) | 14% |
| \| Pituitary \| \| --- \| | 15% (12) | 10% |
| Thyroid | 9% (7) | 15% |
| Total N (%) | 78 (100%) | 240 (100%) |
